# Supplementary material for: Comprehensive Annotation and Functional Exploration of MicroRNAs in Lettuce
Source: Front Plant Sci. 2021 Dec 24;12:781836. doi: 10.3389/fpls.2021.781836 (PMC8739914; doi:10.3389/fpls.2021.781836)
Supplement: Supplementary file 3 [file Data_Sheet_9.ZIP › GSM2230753.plot/Lsa-miRN1732_Lsat_1_v5_gn_9_61640.1_2151_TPlot.pdf]

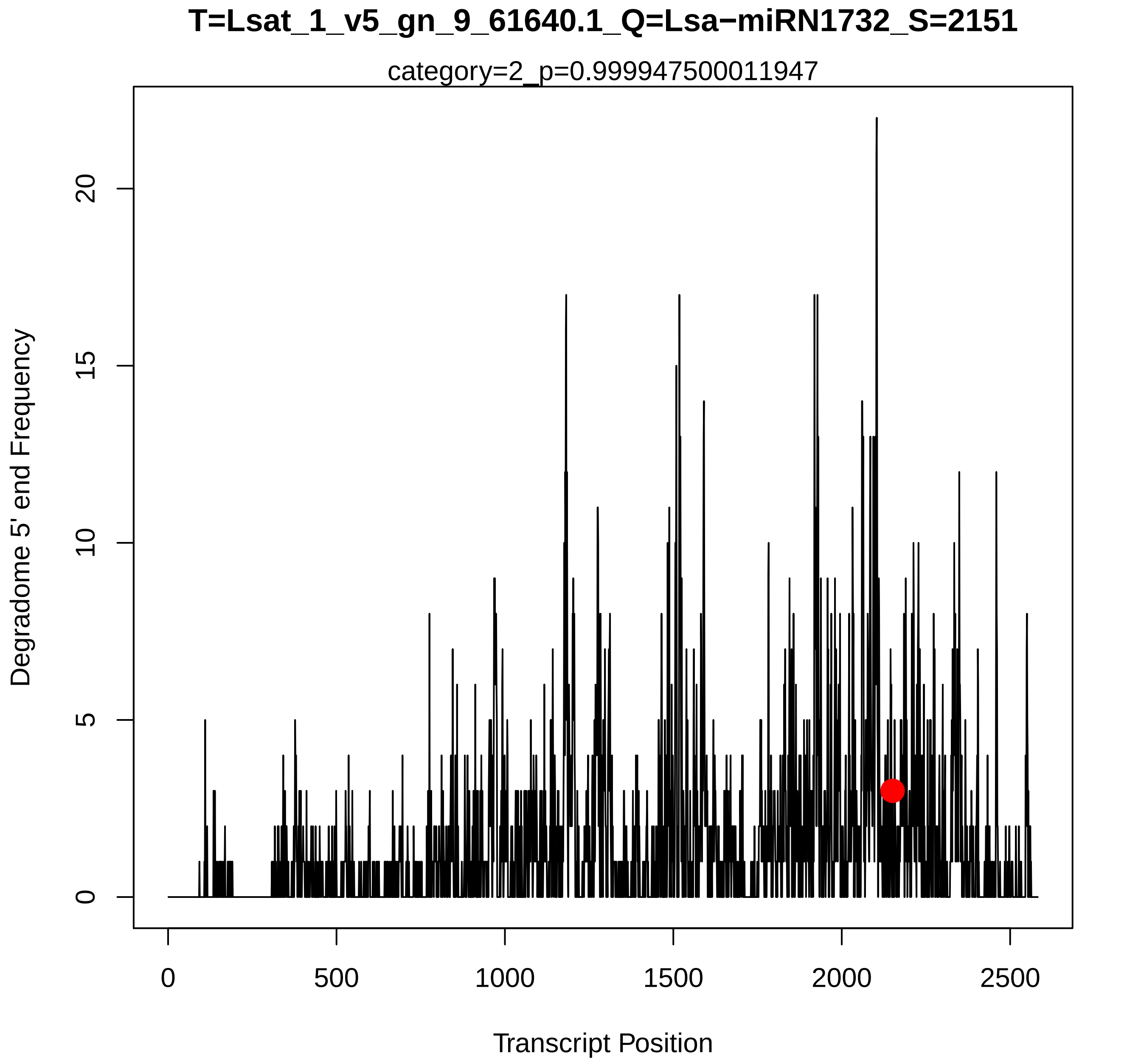
T=Lsat_1_v5_gn_9_61640.1_Q=Lsa−miRN1732_S=2151
category=2_p=0.999947500011947
Degradome 5' end Frequency
0
5
10
15
20
0
500
1000
1500
2000
2500
Transcript Position
